# The osteogenic cell surface marker BRIL/IFITM5 is dispensable for bone development and homeostasis in mice

**DOI:** 10.1371/journal.pone.0184568

**Published:** 2017-09-07

**Authors:** Alexa Patoine, Abdallah Husseini, Bahar Kasaai, Marie-Hélène Gaumond, Pierre Moffatt

**Affiliations:** 1 Shriners Hospitals for Children–Canada, Montreal, Quebec, Canada; 2 Department of Human Genetics, McGill University, Montreal, Quebec, Canada; University of Massachusetts Medical School, UNITED STATES

## Abstract

BRIL (bone-restricted IFITM-like), is a short transmembrane protein expressed almost exclusively in osteoblasts. Although much is known about its bone-restricted gene expression pattern and protein biochemical and topological features, little information is available for BRIL physiological function. Two autosomal dominant forms of osteogenesis imperfecta (OI) are caused by distinct, but recurrent mutations in the *BRIL* gene. Yet, the underlying mechanisms by which those mutations lead to OI are still poorly understood. A previous report indicated that BRIL knockout (KO) mice had bone deformities, shortened long bones, and reproductive problems. Here we generated and systematically analyzed the skeletal phenotype of a new global *Bril* KO/*LacZ* knockin mouse model. KO mice reproduced and thrived normally up to 12 month of age. The skeletal phenotype of KO and WT littermates was assessed at embryonic (E13.5 to E18.5) and postnatal (2 days, 3 weeks, 3 months and 8 months) time-points. Embryos from E13.5 through to E18.5 showed significant X-Gal staining in all skeletal elements without any apparent patterning anomalies. Although bone deformities were never observed at any postnatal ages, minor and transient differences were noted in terms of bone length and static uCT parameters, but not systematically across all ages and genders. These changes, however, were not accompanied by significant alteration in bone material properties as assessed by a 3-point bending test. In addition, no changes were detected in circulating serum markers of bone turnover (P1NP, CTX-I, and osteocalcin). Gene expression monitoring also revealed no major impact of the loss of BRIL. Further, when mice were challenged with a surgically-induced fracture in tibia, bones repaired equally well in the KO mice as compared to WT. Finally, we showed that BRIL C-terminus is not a bona fide binding site for calcium. In conclusion, our in depth analysis suggest that skeletal patterning, bone mass accrual and remodeling in mice proceeded independent of BRIL.

## Introduction

Bone-restricted IFITM-like (BRIL), also known as interferon-induced transmembrane protein 5 (IFITM5), is a small 132 amino acid membrane protein expressed specifically in osteoblasts of bones from both intramembranous and endochondral ossification origins [1, 2]. BRIL is a palmitoylated type II transmembrane protein (N-in/C-out topology) that is present at the plasma membrane and Golgi apparatus of the osteoblast [3, 4]. Based on structural homology and chromosomal localization, BRIL is categorized as a member of the IFITM protein family. However, characteristics and properties of BRIL make it highly unique from other family members, including the bone-specific expression profile of the protein [1, 2] and gene regulation by mechanisms not involving induction by interferons [5, 6]. In contrast to BRIL, other IFITM members (IFITM1, 2, 3) display a generally ubiquitous expression pattern and can be transcriptionally induced by interferons. They are considered one of the first lines of defense against viral infection, blocking cellular entry of diverse viruses [7, 8].

Initial characterization of BRIL *in vitro* indicated that the protein was likely involved with bone formation, given that its peak temporal expression in osteoblasts coincides with matrix production and mineralization of cells [1, 9]. Similarly, *in vitro* overexpression and knockdown assays in MC3T3 and UMR106 osteoblasts suggested a role for BRIL as a positive modulator of mineralization [1]. Other *in vitro* work suggested an alternative function for BRIL in regulating immune system activity in the skeleton [10].

Osteogenesis imperfecta (OI) is a family of bone genetic disorders characterized by brittle bones [11]. Remarkably, in line with its *in vitro* functions, the importance of BRIL in humans was later demonstrated by the identification of mutations in the encoding gene as the causes of two distinct forms of autosomal dominant osteogenesis imperfecta (OI). First, a recurrent heterozygous mutation in the 5’-untranslated region (c. -14C>T) was identified as the single underlying genetic cause of OI type V; whereby this point mutation creates a novel in-frame translation start site that adds a 5 amino acid extension (Met-Ala-Leu-Glu-Pro) onto the N-terminus of BRIL [12, 13]. Additionally, probands have been identified with a *de novo* heterozygous missense mutation in exon 1 of *BRIL* (c. 119C>T; p. Ser40>Leu) which results in a severe progressive atypical OI type VI phenotype [14, 15]. The OI disease causing mutations in *Bril* are believed to result in contrasting neomorphic functions [3, 16], typified on one hand by ectopic mineralization in type V, and on the other by decreased mineralized osteoid in the atypical type VI. Hence, these differing human conditions would not help clarify the underlying mechanism of action and normal activity carried out by BRIL.

Although BRIL expression has been demonstrated in bone tissues from rodents [1, 2, 6], humans [3, 13], tammar wallaby [5], and chicken [17], a clear *in vivo* role for the protein is still largely elusive. One hypothesis put forward is that the acidic C-terminus of BRIL represents a calcium binding moiety, although no formal experimental validation has been performed [18, 19]. Studies by Lietman *et al*. [16] have demonstrated that osteoblast-specific overexpression of BRIL in a transgenic mouse model, under regulation of the collagen type I promoter, resulted in an absence of phenotypic consequences on the skeleton. Additionally, mice generated with the entire locus of the five *Ifitm* genes deleted (*IfitmDel*) do not appear to possess any gross skeletal abnormalities, although this phenotype was not directly characterized [20]. *IfitmDel* mice are comparable to wild-type littermates with regard to their size, weight as well as behaviour at birth and are fertile as adults [20], although this has more recently been questioned with respect to the mouse genetic background [21]. The only formally described phenotypes in these animals are an increased sensitivity to viral infections [7, 8] as well as the development of a moderate metabolic syndrome in adulthood [22]. Finally, a specific global *Bril* knockout (KO) mouse was reported to display a mild skeletal phenotype at birth and persistent through adulthoods, and with potential reproductive problems [2]. Newborn mice displayed shorter skeletons and some bent bones but their trabecular and cortical bone parameters were not significantly altered. Adult *Bril* KO mice (40–51 weeks old) continued to show persistently shorter long bones but not bending. Unfortunately, no static bone morphometric measurements were provided for the adults. Therefore, there is still no unifying information about BRIL function, some even presenting apparently conflicting outcomes.

To investigate more thoroughly the *in vivo* repercussions of the loss of BRIL we generated another mouse *Bril* KO/LacZ knockin, and assessed the skeletal phenotype of animals at multiple time points throughout postnatal life (2 days, 6 weeks, 3 months 8 months). The *Bril* KO had no gross defects and reproduced normally. Under steady state normal vivarium conditions, we took measures of static trabecular and cortical bone formation by uCT, mechanical properties by 3 point bending, and determined blood biochemistry as well as bone turnover markers. Gene expression monitoring was also performed by RT-qPCR. No consistent and systematic differences were detected in any of the parameters tested, even after challenge of the mouse with a tibia fracture repair model. In addition, we provide the first experimental evidence that the BRIL C-terminus is not a formal calcium binding domain, as was previously hypothesized. Altogether, our data indicate that the absence of BRIL does not significantly impact skeletal integrity *in vivo* in mice.

## Materials and methods

### Generation of *Bril* KO mouse model

A targeting vector was constructed and used to create a global conventional gene KO of *Bril* in mice. The targeting strategy, replaced the 2 coding exons of the *Bril* gene by the nuclear localization signal (NLS)-tagged LacZ (NLS-LacZ) and PGK-Neo cassettes configured in a tail to tail orientation. The 2 homology arms consisted of a 3.9kb fragment upstream of the natural translation start site (iMET), and a 5kb fragment covering intergenic region downstream of exon2. The identity of the final construct was confirmed by Sanger sequencing on an Applied Biosystem 3730xl DNA Analyzer through the McGill University and Genome Quebec Innovation Centre. A NotI linearized construct (11.9kb) was electroporated into R1 ES cells using the services of the Goodman Cancer Center Transgenic core facility at McGill University. G418 resistant clones were screened by Southern blotting with the indicated 5’ probe, which lies outside the targeting construct. In brief, cells in 96-well plates were treated with proteinase K (0.5mg/ml in Tris-HCl 10mM (pH7.5), EDTA 10mM, Sarkosyl 0.5% w/v)) and incubated at 55°C overnight. The gDNA was precipitated with 75mM NaCl in 100% ethanol, washed with 70% ethanol, then digested with SpeI. The digested gDNA was analyzed by 1% agarose gel electrophoresis, transferred to nylon positively charged membrane, and probed by Southern blot with a [α-^32^P]dCTP (3000 Ci/mmol) (PerkinElmer Life Sciences) random primed-labeled 5’ probe. Positive ES clones were selected, expanded in 6-well plates, and reanalyzed to confirm their identity. Positive mutant ES cell clones were injected into blastocysts by the transgenic core facility, and then implanted into pseudo-pregnant females to generate chimeric mice. The animal use protocol and all procedures were reviewed and approved by the Shriners Hospitals for Children Animal Care Committee and the McGill Institutional Animal Care and Use Committee. Shriners Hospitals for Children–Canada and McGill University are accredited and followed the guidelines of the Canadian Council on Animal Care. All animals used (n = 256) in subsequent analyses have a mixed genetic background (C57BL/6x129Sv).

### PCR genotyping

Genotyping was performed using PCR amplification on gDNA prepared from mouse tail tissue. Briefly, mouse tail clippings were digested overnight at 55°C in lysis buffer (0.1 mM Tris (pH 8), 0.2 M sodium chloride (NaCl), 5 mM ethylenediaminetetraacetic acid (EDTA), 0.4% sodium dodecyl sulfate (SDS)) with 250 μg proteinase K (Fisher Bioreagents). DNA was precipitated using one volume of isopropanol, washed with 70% ethanol and was re-suspended in Tris-EDTA buffer (10 mM Tris (pH 8) and 0.1 mM EDTA). A standard PCR mix was utilized for gDNA amplification including 1x standard Taq buffer, 5 mM dNTP mix and 1.75 units of Taq DNA polymerase (all New England Biosystems) with 12.5 μM of primers. A single forward primer (5’-GAAGTAGAGAGAGCAGCG-3’) was used in combination with primers hybridizing to either the wild type (WT) allele (5’-GTGTAGGGCCAGGTGTTCC-3’) or the KO/LacZ-KI sequence (5’-CTTTCTCTTCTTCTTCGGCCC-3’). The PCR products (WT = 366bp; KO = 287bp) were resolved on 1.5% agarose gel.

### RNA and protein extractions

RNA and proteins were extracted from bones utilizing TRIzol reagent (Life Technologies), according to the manufacturer’s instructions. Resulting protein pellets were re-suspended in NP-40 (nonyl phenoxypolyethoxylethanol) cell lysis buffer (50 mM Tris-HCl (pH 7.4), 150 mM NaCl, 1 mM EDTA, 1% (v/v) NP-40) with 1% protease cocktail inhibitor (Sigma) and were then sonicated for 5 minutes utilizing a Bransonic 12 Ultrasonic Cleaner (Branson Ultrasonics Corporation). Extracted proteins were combined with 4x Laemmli buffer (200 mM Tris-HCl pH 6.8, 8% SDS, 40% glycerol, 50 mM EDTA, 0.08% bromophenol blue) with 5% β-mercaptoethanol, boiled for 10 minutes and then stored at -20°C until analysis. Proteins were also extracted from 6w old liquid nitrogen snap frozen half calvaria directly in NP-40 cell lysis buffer with 1% protease inhibitor cocktail utilizing a Tissumizer Tissue Homogenizor. Total tissue homogenates were then centrifuged at 16000g for 10 minutes at 4°C. Soluble proteins from the homogenate supernatant were collected and combined with 4x Laemmli buffer with 5% β-mercaptoethanol, boiled for 5 minutes and stored at -20°C until analysis.

### Western blotting

Protein samples were loaded and separated on 1.5 mm NuPAGE 4–12% Bis-Tris Protein Gels (Novex) in NuPAGE MES (2-(N-morpholino)ethanesulfonic acid) Running Buffer (Novex). Proteins were transferred to 0.45 μm nitrocellulose membranes (Protran BA85) and stained with Ponceau S red to assess lane protein content, migration and transfer. Membranes were blocked for 1 hour in 5% skim milk/PBS-tween (0.05%) then blotted overnight at 4°C in primary antibody (affinity purified rabbit anti-mouse N-terminal BRIL [1, 3, 6] at a 1/4000 dilution in 5% skim milk/PBS-tween (0.05%)). Membranes were then blotted in HRP-coupled goat anti-rabbit secondary antibody (Amersham) at a 1/30000 dilution in 5% skim milk/PBS-tween (0.05%) for 1 hour at room temperature. Detection was performed with ECL Prime Western Blotting Detection Reagent (Amersham).

### β-galactosidase staining and enzymatic assay

Mouse embryos were collected at specified developmental stages from staged pregnant females, day 0.5 denoting the morning of vaginal plug detection and processed as described [23]. Embryos were fixed (1% PFA, 0.25 mM ethylene glycol tetraacetic acid (EGTA), 0.2% (v/v) NP-40, 0.1 mM magnesium chloride (MgCl_2_), 0.2% glutaraldehyde) and processed for either cryosectioning followed by β-galactosidase staining or whole mount β-galactosidase staining. For cryosectioning, embryos were washed post-fix in 1x PBS then incubated in 20% sucrose for 24 hours at 4°C followed by 24 hours in 1:1 20% sucrose:OCT (Clear Frozen Section Compound, VWR) at 4°C. Tissue samples were embedded in 1:1 20% sucrose:OCT by flash freezing on dry ice and were stored at -20°C until sectioning. Cryosection blocks were cut at a thickness of 10 μm utilizing a Bright Model OTF Cryostat (Instrument Company Ltd.). For staining cryosections, slides were first fixed in 0.2% glutaraldehyde/PBS for 10 minutes at room temperature. Sections were washed with LacZ wash solution (1 mM MgCl_2_, 0.01% deoxycholate, 0.02% (v/v) NP-40) and then incubated in LacZ stain (0.25 mM potassium ferricyanide (K3), 0.25 mM potassium ferrocyanide (K4), 50 mg/mL X-Gal (5-bromo-4-chloro-3-indolyl-β-D-galactopyranoside) in LacZ wash solution) at 37°C for 2–4 hours. Sections were subsequently counterstained for 30 seconds with 1% eosin Y (w/v in Milli-Q water) then washed in distilled water, dried and mounted. Another set of X-Gal stained consecutive sections were counterstained for 10 minutes with picrosirius red (Direct Red 80 (Sigma-Aldrich) at 1 mg/mL in 1.3% saturated picric acid). After staining, slides were washed with two changes of 0.5% acetic acid, rinsed with distilled water, dried and amounted. For whole mount β-galactosidase staining, embryos were washed post-fix in LacZ wash solution and then incubated for 3.5 hours in LacZ stain solution at 37°C with end-over-end rotation. After staining, embryos were fixed for 24 hours in 3% PFA/PBS at 4°C then dehydrated to 70% ethanol for storage at 4°C. The Tropix Galacton-Plus chemiluminescent enzyme assay kit (Applied Biosystems) was used to quantify b-galactosidase activity from whole bone tissue extracts. Bones were homogenized using the kit solubilisation buffer and assayed with the Galacton-plus substrate in a Sirius luminometer (Berthold, Oak Ridge, TN). Activity was normalized to total protein content as measured by the Bradford Protein Assay (Bio Rad).

### Whole skeleton staining with alizarin red and alcian blue

Two day old pups were processed for the whole skeleton staining with alizarin red (bone) and alcian blue (cartilage) according to standard procedures [24, 25].

### Skeletal morphometry

Micro-computed tomography (μCT) imaging was performed to characterize trabecular and cortical bone parameters. Briefly, the PFA-fixed and dehydrated left femur of 6w, 3m, and 8m old animals (n = 10; 5 males and 5 females) were scanned *ex vivo* with soft tissue removed. Scans were conducted using a 5 μm pixel size in a SkyScan/Bruker 1172 high-resolution μCT scanner located at the McGill University Health Center Orthopedic Research division (C9). After scanning, raw femur images were reconstructed using the Skyscan NRecon program with a beam hardening correction of 30, with a ring artifact of 4 and dynamic range values of 0 and 0.13. Trabecular and cortical bone microarchitecture were analyzed using the Skyscan CT Analyzer program.

For the trabecular bone analysis, the region of interest (ROI) was defined manually by contouring an irregular anatomical region, a few pixels from the endocortical surface, every 5 bone slices. The ROI contouring began after an offset of 0.845 mm from the growth plate and extended proximally 1.27 mm towards the midshaft for 6w old animals, began after an offset of 0.25 mm from the growth plate and extended 1.25 mm towards the midshaft for 3m old animals and began after an offset of 0.195 mm from the growth plate and extended 1.25 mm towards the midshaft for 8m old animals. To define bone, an upper threshold of 255 was used with a lower adaptive threshold of 65 for 6w old bones or a lower adaptive threshold of 70 for 3 and 8m old bones. From these defined ROIs, three-dimensional measurements were calculated for bone volume fraction (bone volume/tissue volume, %), trabecular thickness (mm), trabecular separation (mm) and trabecular number (1/mm).

For the cortical bone analysis, an offset of 4.425 mm proximal from the growth plate at 6w old, an offset of 5.135 mm proximal from the growth plate for 3m old and an offset of 6 mm proximal from the growth plate at 8m old was used as a starting point to define a ROI of 1.003 mm in the diaphysis to conduct measurements on. To define bone, an upper threshold of 255 and a lower global threshold of 85 were utilized for samples at all age points. From these defined ROIs, two-dimensional measurements were calculated for total cross-sectional tissue area (mm^2^), total cross-sectional bone area (mm^2^), bone area fraction (bone cross-sectional area/tissue cross-sectional area, %) and cross-sectional thickness (mm). Prior to scanning, the length of each femur was measured from the top of the medial condyle to the bottom of the major trochanter utilizing the preview x-ray image.

### Biomechanical testing

The three point bending assay was used to characterize biomechanical properties of 3m and 8m old bones. The right femur was cleaned of soft tissue and loaded to failure at room temperature in the anterior to posterior direction using an Instron model 5943 single column table frame machine with a loading rate of 0.05 mm/s and a support distance of 7 mm. From the generated load vs. displacement curve measurements were taken for: the slope of the initial linear region (stiffness, N/mm), maximum load (N), load at break (N), area under the load-displacement curve (energy to failure, N·mm) and displacement at break (maximum deformation, μm).

### Real-time quantitative polymerase chain reaction (RT-qPCR)

For RT-qPCR, cDNA was first prepared utilizing 2 μg of prepared humerus RNA with the High Capacity cDNA Reverse Transcription Kit (Applied Biosystems), according to the manufacturer’s instructions for 20 μl reactions without an RNase inhibitor. Real-time PCR reactions of 25 μl were set up with 1 in 5 diluted cDNA in triplicate with MicroAmp Optical 96-Well Reaction Plates utilizing TaqMan Universal PCR Master Mix and TaqMan Gene Expression Assay probes *(β-actin*: Mm00607939_s1; *Bril*: Mm00804741_g1; *Alpl* (alkaline phosphatase tissue non-specific): Mm00475834_m1; *Bglap* (osteocalcin): Mm03413826_mH; *Col1a1* (collagen type 1 alpha 1): Mm00801666_g1; *Catk* (cathepsin K): Mm00484039_m1; *Sost* (sclerostin): Mm04208528_m1; *Osx*: Mm00504574_m1) according to the manufacturer’s instructions (all supplies/reagents Applied Biosystems). RT-qPCR was run on a 7500 Real Time PCR System (Applied Biosystems) utilizing the Relative Quantification plate assay function of the 7500 System Sequence Detection Software (SDS) v.1.3.0. All data were normalised to β-actin and values were expressed as 2^(-ΔCt)^ [26].

### Serum biochemistry

Blood was collected in BD Microtainer Tubes. After 30 minutes at room temperature, samples were spun at 9000 rpm for 1 minute to separate blood components. Serum of 6w and 3m old animals was analyzed with enzyme linked immunosorbent assays (ELISA) to measure C-terminal telopeptides of type I collagen (CTX-I) with the RatLaps EIA (Immunodiagnostic Systems) as well as measure N-terminal propeptides of type I collagen (P1NP) with the Rat/Mouse P1NP EIA (Immunodiagnostic Systems) to assess bone resorption and formation, respectively. Assays were performed and analyzed according to manufacturer’s instructions; except with a 1 in 4 dilution of 6w old serum samples for analysis of CTX-I by ELISA. The ELISA kits for measuring the carboxylated (Gla-OCN) and undercarboxylated (Glu-OCN) forms of osteocalcin in serum were purchased from Takara. A complete serum biochemistry workout, measuring 16 analytes, was also performed at the McGill University Comparative Medicine & Animal Resources Centre Diagnostic Laboratory on a Vitros 250 apparatus (Ortho Clinical Diagnostics).

### Bone length measurements, x-ray imaging, and histology

The length of PFA-fixed femurs was measured by μCT x-ray imaging (see below). High resolution X-ray images were captured on a Faxitron MX-20 instrument at the McGill Centre for Bone and Periodontal Research. After imaging, samples were processed for methylmethacrylate embedding and sectioning. Consecutive sections were de-plasticized with 4 consecutive 20 min baths in ethylene glycol monoethyl ether acetate (Fisher), and rehydrated to H_2_O through descending ethanol baths. Staining for mineralized (green) and un-mineralized osteoid (pink) was performed with Goldner, and von Kossa was used to stain for calcium (black). Toluidine blue was used to counterstain the von Kossa slides. Tibias from the fracture repair surgeries were decalcified for 3w at 4C with 15% EDTA (w/v H_2_O; pH7.2), changing for fresh EDTA every 3-days. Sample were dehydrated to ethanol 70% and processed for paraffin embedding. Six μm thick sections were deparaffinized with xylenes, re-hydrated through descending ethanol baths, and processed for histological assessment of collagen deposition using picrosirius red and cartilage proteoglycans using alcian blue. Briefly, staining was performed for 1h at room temperature either with sirius red (Direct red 80) 0.1% (w/v) in saturated aqueous picric acid, or with alcian blue 8GX 0.5% (w/v) in 0.1M HCl. Excess staining solution was rinsed off with several times with 0.5% acetic acid or distilled H_2_O. Alcian blue slides were counterstained with nuclear fast red.

### Rodded fracture repair surgeries and analysis

An open-type osteotomy technique to simulate intramedullary tibial nailing in 4m mice was adapted from published procedures [27, 28]. Surgeries were performed aseptically under isoflurane anesthesia. Mice received dual mode analgesia using carprofen (10 mg/kg) and slow release buprenorphine (1 mg/kg), administered SC 30 min pre-op. Animals were monitored twice daily thereafter, and carprofen was re-administered if needed. After shaving the leg, a 3mm vertical incision was made in the skin above the knee and the patellar ligament was exposed. Using a #11 blade scalpel, a 2mm medial parapatellar incision was performed and the knee joint space was exposed. Through the newly formed space, a 26G needle was inserted along the direction of the long axis of the tibia, through the plateau into the canal. Through the needle, the internal wire core guide of a 25G spinal needle (Quincke 25G 3” spinal needle, BD #450170, Franklin Lakes, USA) was inserted and the 26G needle was then removed while keeping the internal wire core guide in the tibial canal. The wire was then cut at the level of the tibial plateau and bent at 90 degrees at its proximal part to avoid puncture of the patellar ligament. Using extra fine Bonn scissors (Fine Science Tools #14084–08, Vancouver, Canada), an osteotomy was performed to simulate a tibial mid shaft transverse fracture. Two weeks after the procedure, mice were euthanized and tibiae were collected by disarticulating the tibia at the level of the knee and the ankle. The intramedullary nail was carefully pulled out using a needle holder and the tibiae were fixed for 24h in 4% PFA at 4°C. After washing with PBS, samples were dehydrated to 70% ethanol, and imaged by μCT as described above. All scans were made using the Skyscan 1.5 software at a source voltage of 65kV and a source current of 153 μA. A medium camera setting with an aluminum filter was used. The samples were then scanned in two parts at an image pixel size of 4.84 μm. For every 0.45 degrees of sample rotation, 3 image frames were retained. A total vertical segment of 9mm was scanned for every sample generating around 900 consecutive transverse image slices. The obtained images were then analyzed using the SkyScan CTan 1.11 software. The region of interest for analysis was selected to include included 2mm above and 2mm below the osteotomy site, corresponding to a rectangular volume of 4mm x 2mm x 2mm. The gray scale used to measure bone inside this volume was set to have a minimum of 55 and a maximum of 255. This allowed us to obtain BV/TV percentages that could be compared to each other based on the common volume of interest.

### Calcium binding assay

The mouse *Bril* and calmodulin 1 (*Calm*) coding sequences were cloned in the isopropyl β-D-1-thiogalactopyranoside (IPTG)-inducible pQE30 bacterial expression plasmid (QIAGEN). Both bacterial proteins were expressed with 6-histidine tag fused at their N-termini. Mouse CALM served as a positive control and its cDNA was first amplified by RT-PCR on RNA isolated from MC3T3 osteoblasts using forward (5’-CTTCCTTCGCTCGCACCATGG-3’) and reverse primers (5’-CTTCATTTTGCAGTCATCATCTG-3’). Bacterial cultures were grown to log phase, induced with 1mM IPTG for 4h and pelleted. Total protein extracts were prepared from bacterial pellets by sonication in 50mM NaH_2_PO_4_ buffer (pH8). Lysates were cleared by centrifugation at 10,000g for 15min 4C. Soluble proteins in the supernatant were mixed with 4X Laemmli sample buffer containing β-mercaptoethanol, and separated on 8–16% gradient SDS-PAGE (BioRad). Proteins were transferred to PVDF membranes and assayed for protein expression by western blotting with a monoclonal anti-histidine antibody (clone His.H8; Millipore 05–949), or for ^45^CaCl_2_ (PerkinElmer) binding essentially as described [29]. Briefly, blots were washed 3-times 20min with 10mM imidazole-HCl buffer (pH6.8) containing 60mM KCl and 5mM MgCl_2_. Binding was performed for 30min at room temperature in 20mL imidazole-HCl wash buffer supplemented with 50μCi of ^45^CaCl_2_. Blots were rinsed 3-times with distilled H_2_O, once for 5min, and then for 5min with 50% ethanol. Calcium binding proteins were detected by exposure of the dried blots to autoradiographic films for 15h at room temperature.

### Statistical analyses

Data is expressed as mean ± standard deviation (SD) or SEM, as indicated. Significance was assessed utilizing ANOVA followed by Bonferroni post-hoc test, or a two-tailed unpaired t-test with a p-value ≤ 0.05 being considered statistically significant (*<0.05; **<0.01; ***<0.001). Statistical analyses were performed utilizing GraphPad Prism 5 (GraphPad Software).

## Results

### Generation of and beta-galactosidase activity in *Bril* KO mice

**Fig 1:**
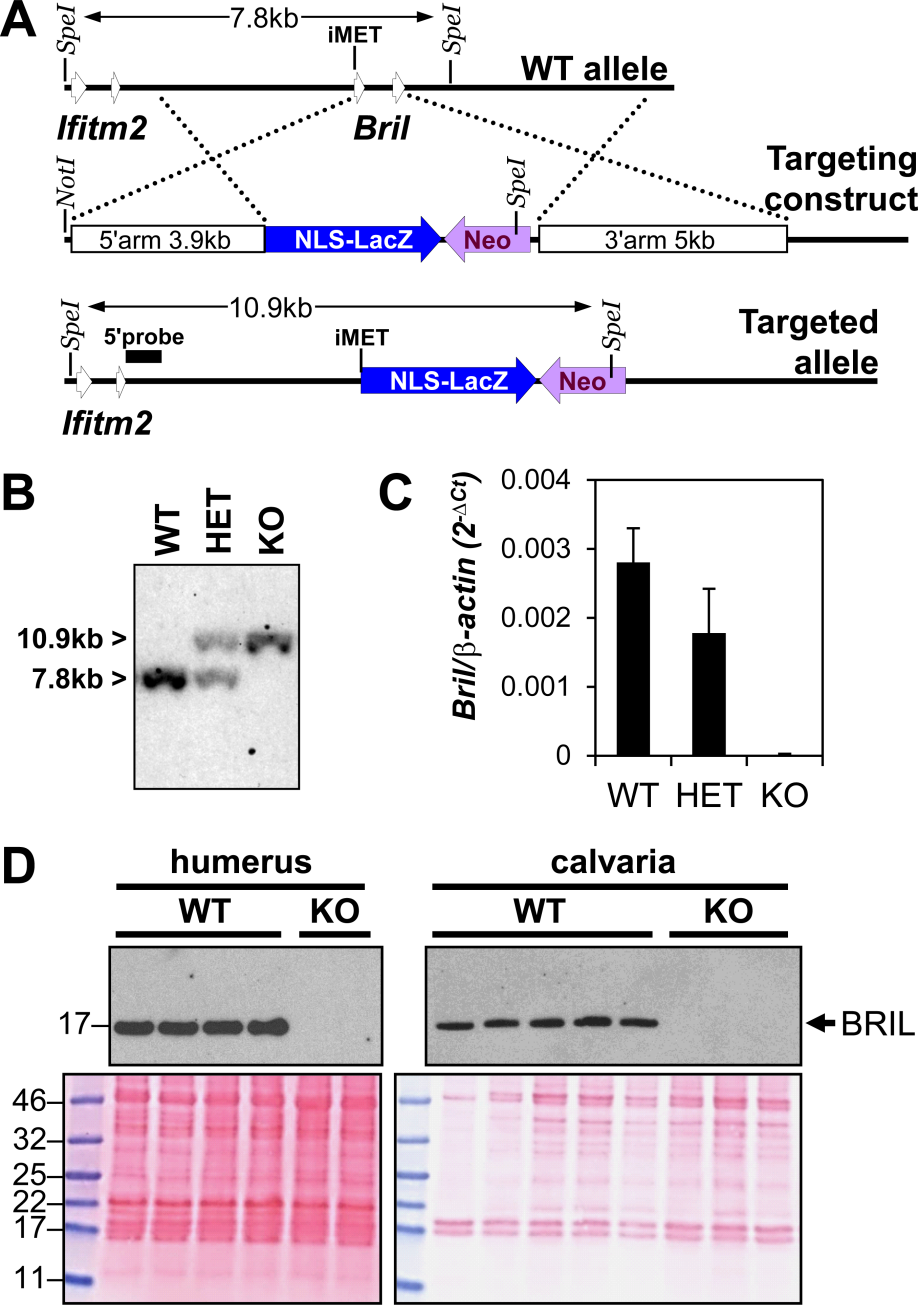
Generation and validation of the *Bril* KO;*LacZ* knockin mouse model. A) Schematic representation of the strategy used for targeting the *Bril* gene. B) Representative Southern blot analysis of genomic DNA, restricted with SpeI, from wild type (WT), heterozygote (HET), and homozygote *Bril* KO mice. C) *Bril* expression as determined by RT-qPCR on total RNA extracted from humeri of 6w old WT, HET and KO mice. D) Western blot analyses of BRIL in proteins extracted from 6w old humeri (WT n = 4, KO n = 2) or calvaria (WT n = 5, KO n = 3). Ponceau S stained membranes (lower panels) are presented as a loading control.

The strategy utilized to generate the *Bril* KO mice is depicted in Fig 1A. For this purpose, a targeting vector containing 5’ and 3’ homology arms of 3.9 and 4.1kb, respectively, was constructed and used to create a global conventional gene KO of the *Bril* gene. The targeting strategy replaced the 2 coding exons of *Bril* by a nuclear localization signal (NLS)-tagged LacZ (NLS-LacZ) and the pGK-Neo cassette. The construct was electroporated into R1 129sv ES cells, G418 clones were screened for an adequate recombination event, and used for chimera generation by blastocyst injection. Chimera were mated with WT C57bl6 and descendants screened by Southern blot analysis which revealed the recombination events had occurred as anticipated (Fig 1B). RT-qPCR analyses confirmed that the gene had been successfully inactivated (Fig 1C), as no transcript was detected in 6w old *Bril* KO humeri. A slight but non-statistically significant reduction of *Bril* expression was noted in the heterozygote (HET). Western blotting also demonstrated the absence of any BRIL protein in both the humerus and calvaria (Fig 1D) as compared to the WT littermates. All animals used in subsequent analyses have a mixed genetic background (C57BL/6x129Sv).

**Fig 2:**
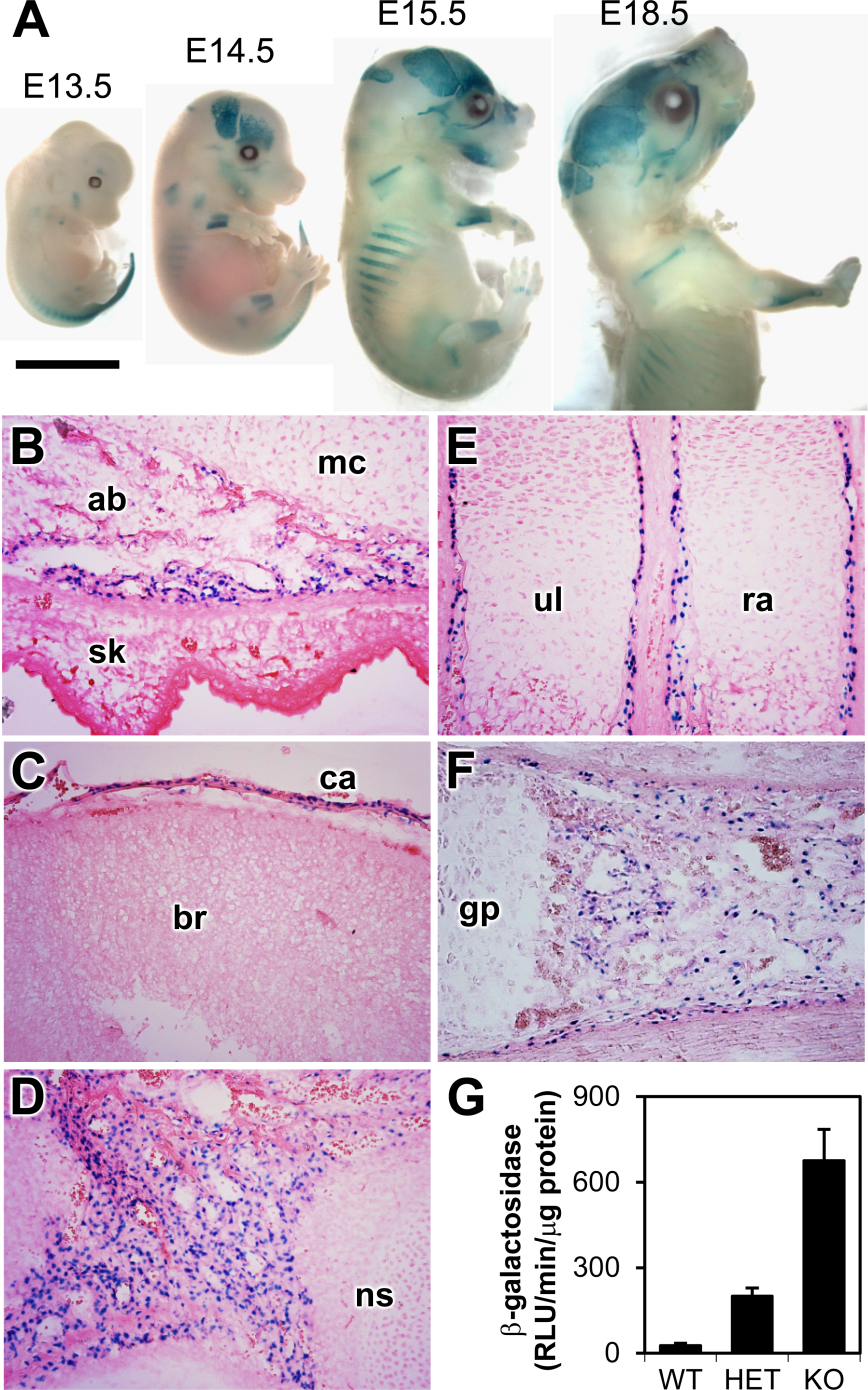
Monitoring of the activity of the *Bril* knockin *LacZ* cassette. A) Whole mount X-Gal staining of *Bril* KO embryos at stage E13.5, E14.5, E15.5, and E18.5 (scale bar = 0.5mm). B-F) Due to the NLS-LacZ cassette, X-Gal staining on cryosections shows nuclear staining in E15.5 mandible (B), calvaria (C), orbital bone (D), forearm (E), and E17.5 femur (F). G) Quantitative activity of β-galactosidase measured in calvaria extracts of 9w old WT, HET, and KO mice. **ab**: alveolar bone; **br**: brain; **ca**: calvaria; **gp**: growth plate; **mc**: Meckel cartilage; **ns**: nasal septum; **ra**: radius; **sk**: skin; **ul**: ulna.

Because the *Bril* KO mice were visually indistinguishable from wildtype and heterozygote littermates (described next), we first tested the functionality of the knocked-in LacZ reporter gene to monitor for sites of *Bril* expression. Whole embryos at various stages of embryonic development (E13.5, E14.5, E15.5, E18.5) were dissected and processed for whole mount X-Gal staining (Fig 2A). In *Bril* HET and KO embryos, X-Gal staining (blue) was evident at many sites of endochondral and intramembranous bone formation whereas WT present no specific staining. X-Gal staining was first detected at E13.5, presenting a diffuse staining in the tail region, and a specific slight staining in the bony rudiment in the mandible and limbs (Fig 2A). Starting from E14.5 onwards, intense staining was visible in parietal and interparietal regions of forming calvaria, mandible, midshaft portion of the forelimbs and hindlimbs, ossified portion of the rib cage, and vertebrae (Fig 2A). Whole E15.5 and E17.5 embryos were then processed for cryosectioning and X-Gal staining in order to get a more accurate cell-specific distribution and localization (Fig 2B–2F). As expected from the NLS-LacZ cassette, numerous blue nuclei were visible only over bony rudiments, while all surrounding tissues were devoid of specific X-Gal staining. Strong labeling was also observed in the head, the mandible (Fig 2B), calvaria (Fig 2C), and orbital bone (Fig 2D), which forms through the direct conversion of mesenchymal progenitors into osteoblasts. In long bones of limbs, heavy staining was observed in osteoblasts lining the cortical bony collar adjacent to the growth plate at E15.5 (Fig 2E), and within the primary ossification centers, more evident at E17.5 (Fig 2F). Within bones, no other cell types produced specific X-Gal staining including growth plate chondrocytes and marrow cells. To further validate that most positive cells are juxtaposed to the forming mineralizing bone surface, a co-staining procedure with X-Gal followed by picrosirius red counterstaining (marking the extracellular collagen matrix network in dark red) was also performed on similar sections to those presented in Fig 2 (S1 and S2 Figs). Most, if not all, blue nuclei stained cells are in proximity or situated directly over the mineralizing matrix in the E15.5 head (S1 Fig) and E17.5 leg bones (S2 Fig). Weak X-Gal staining was also detected in the nostril area and in the incisor pulp/odontoblasts region (S1 Fig). A quantitative assessment of beta-galactosidase activity performed on 9w old calvarial protein extracts still showed considerable activity in the Het and KO samples (Fig 2G). These results indicated that the LacZ reporter activity recapitulated endogenous *Bril* expression, and closely matched the immunohistological localization of endogenous BRIL protein we have described previously in WT mice [1].

### Gross anatomical parameters and histological analyses of *Bril* KO mice

**Fig 3:**
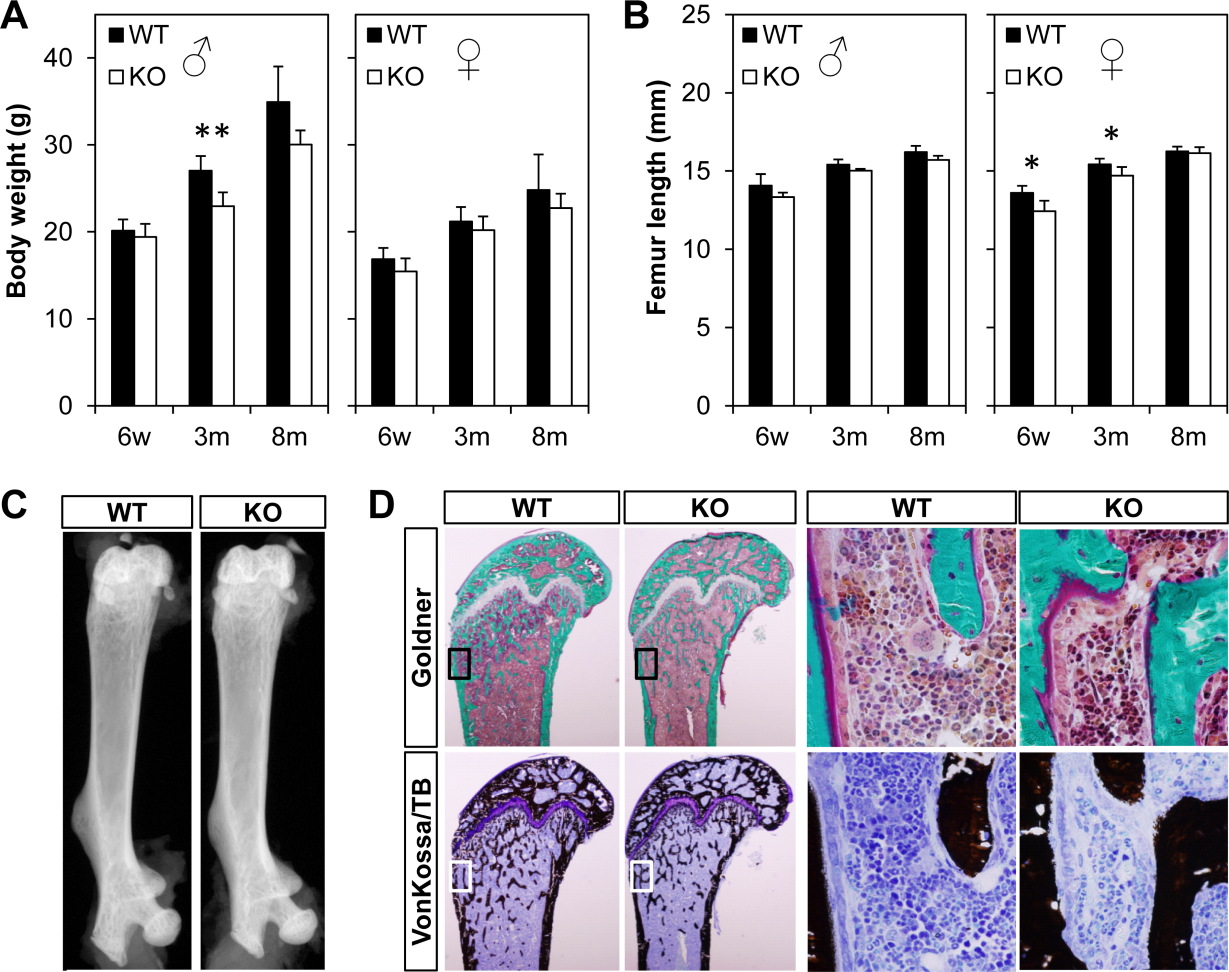
Gross phenotypic analysis of *Bril* WT, HET, and KO mice at age 6w, 3m and 8m. A) Body weight measurements at time of euthanasia for males (left) and females (right). B) Femur length measured using x-ray imaging for males (left) and females (right). A, B) Values represent mean ± SD (n = 3). C) Representative Faxitron x-ray images of WT and KO male mice at 9w. D) Representative histological of non-decalcified femur sections from 9w males. Goldner (green/pink) and vonKossa (black) staining images were captured at low (left) and high (right) magnification (boxed areas). VonKossa counterstain is toluidine blue.

As mentioned above, *Bril* KO mice appeared grossly indistinguishable from littermates at birth and throughout life, up to more than 8m of age. No gross skeletal abnormalities were visible and no spontaneous fractures were observed. Intercross of *Bril* HET mice yielded all 3 genotypes (WT 29%, HET 47%, KO 24%) not significantly differing from the expected Mendelian ratio (25%, 50%, 25%). These numbers were computed from a total of 20 litters with an average size of 7.2 ± 2.1 pups. Interbreeding of *Bril* KO mice also produced comparable size litters (7.0 ± 1.0 pups; n = 12) without any skew in gender number. Body weight (Fig 3A) and femur length (Fig 3B) for males and females were recorded at post-natal ages 6w, 3m, and 8m. Although small statistically significant differences were observed for some parameters, they were not systematically observed across all ages and gender. For example, male KO displayed 15% decreased body weight only at 3m with no changes in femur length. *Bril* KO females present no significant changes in body weight, but had a significant 9% and 4.5% reduction in femur length at 6w and 3m, respectively. Faxitron X-ray images captured for 9w old WT and KO females showed similar anatomical structures and bone density (Fig 3C). Histological staining (Goldner and VonKossa) performed on non-decalcified 9w old femurs revealed no gross changes in bone morphometry, structure, and mineralization of trabecular and cortical elements (Fig 3D). Also, the growth plate architecture was not substantially different between WT and KO mice. Static histomorphometry performed on trabecular bone of 9 week-old males (n = 4) revealed only statistical differences in OV/BV and Ob.S/OS parameters, suggesting higher bone formation (or turnover) activity (S1 Table). Whole body alizarin red and alcian blue staining of 2-day-old pups revealed no striking differences in bone shape, size, or mineralization (S3 Fig).

### μCT assessment of trabecular and cortical bone mineral density and 3-point bending

**Fig 4:**
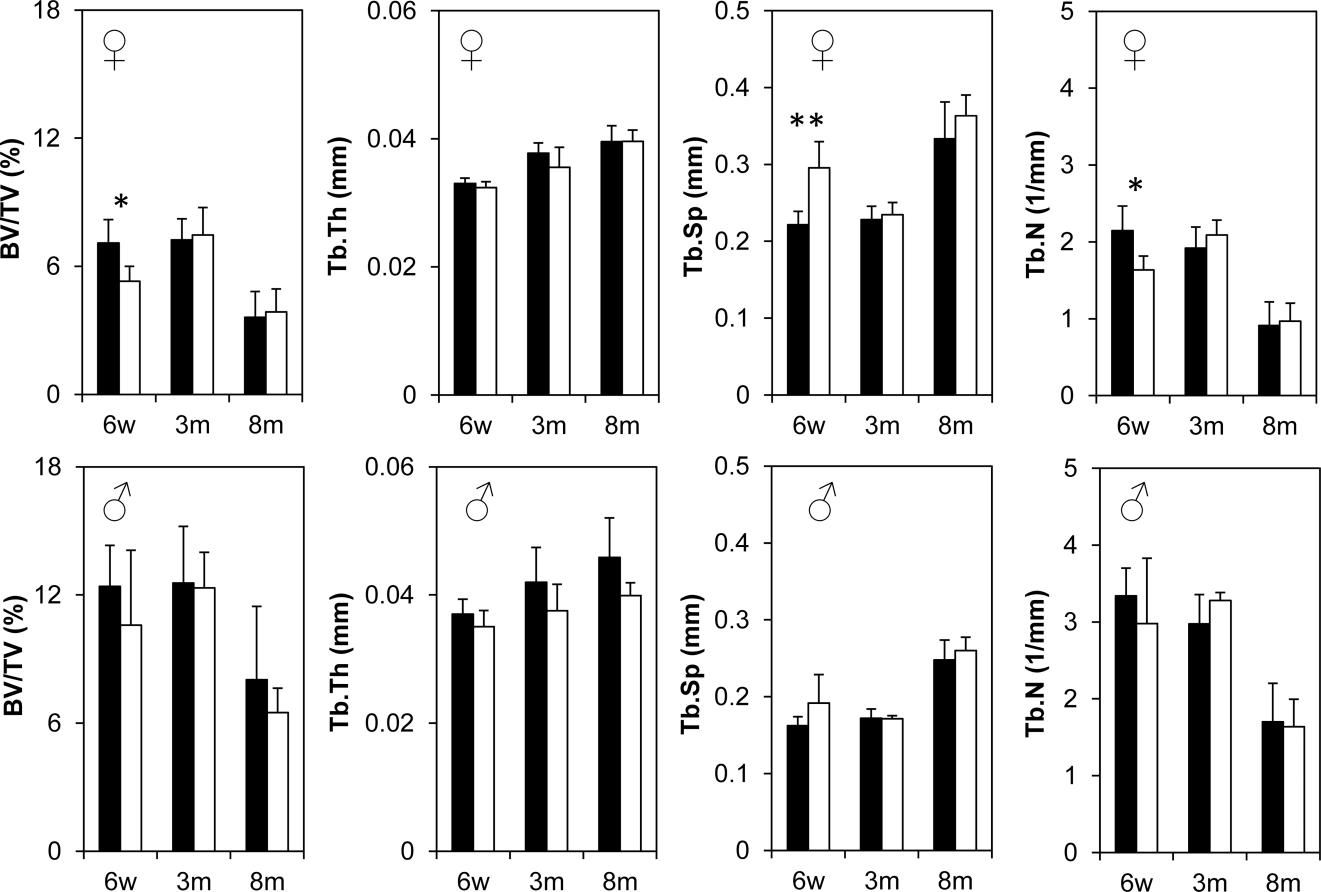
Trabecular μCT analysis of 6w, 3m, and 8m old female (top row) and male (bottom row) distal femurs. Black bars represent WT groups and white bars represent KO groups. BV/TV = bone volume fraction, Tb.Th = trabecular thickness, Tb.Sp = trabecular separation and Tb.N = trabecular number. Values represent mean ± SD at each age point. Group size is n = 5 for all age points except 3m and 8m old KO groups where n = 3.

**Fig 5:**
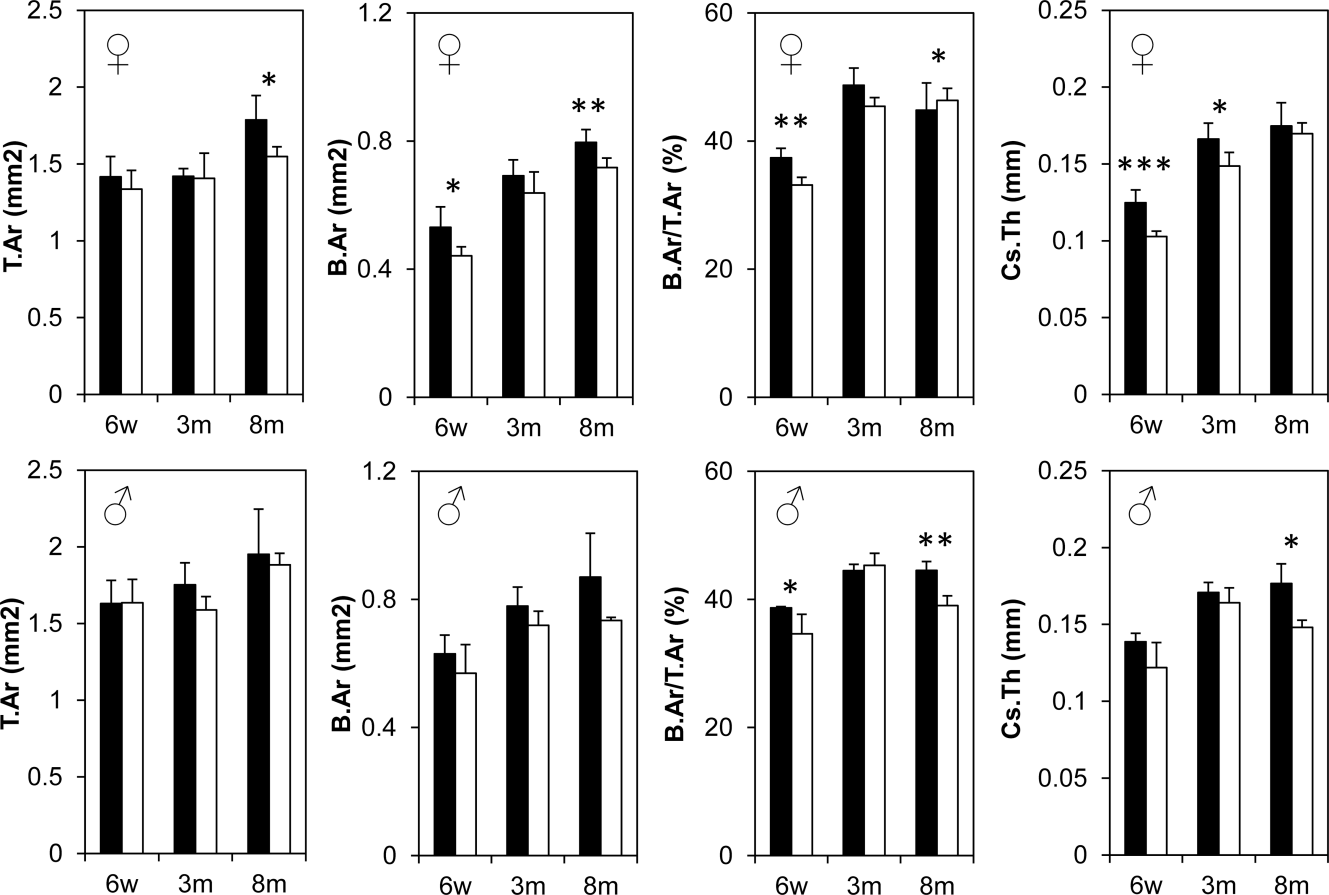
Cortical μCT analysis conducted on 6w, 3m and 8m old female (top row) and male (bottom row) distal femurs. Black bars represent WT groups and white bars represent KO groups. Values represent mean ± SD at each age point. T.Ar = total cross-sectional tissue area, B.Ar = total cross-sectional bone area, B.Ar/T.Ar = bone area fraction and Cs.Th = cross-sectional thickness. Group size is n = 5 for all age points except 3m and 8m old KO groups where n = 3.

**Fig 6:**
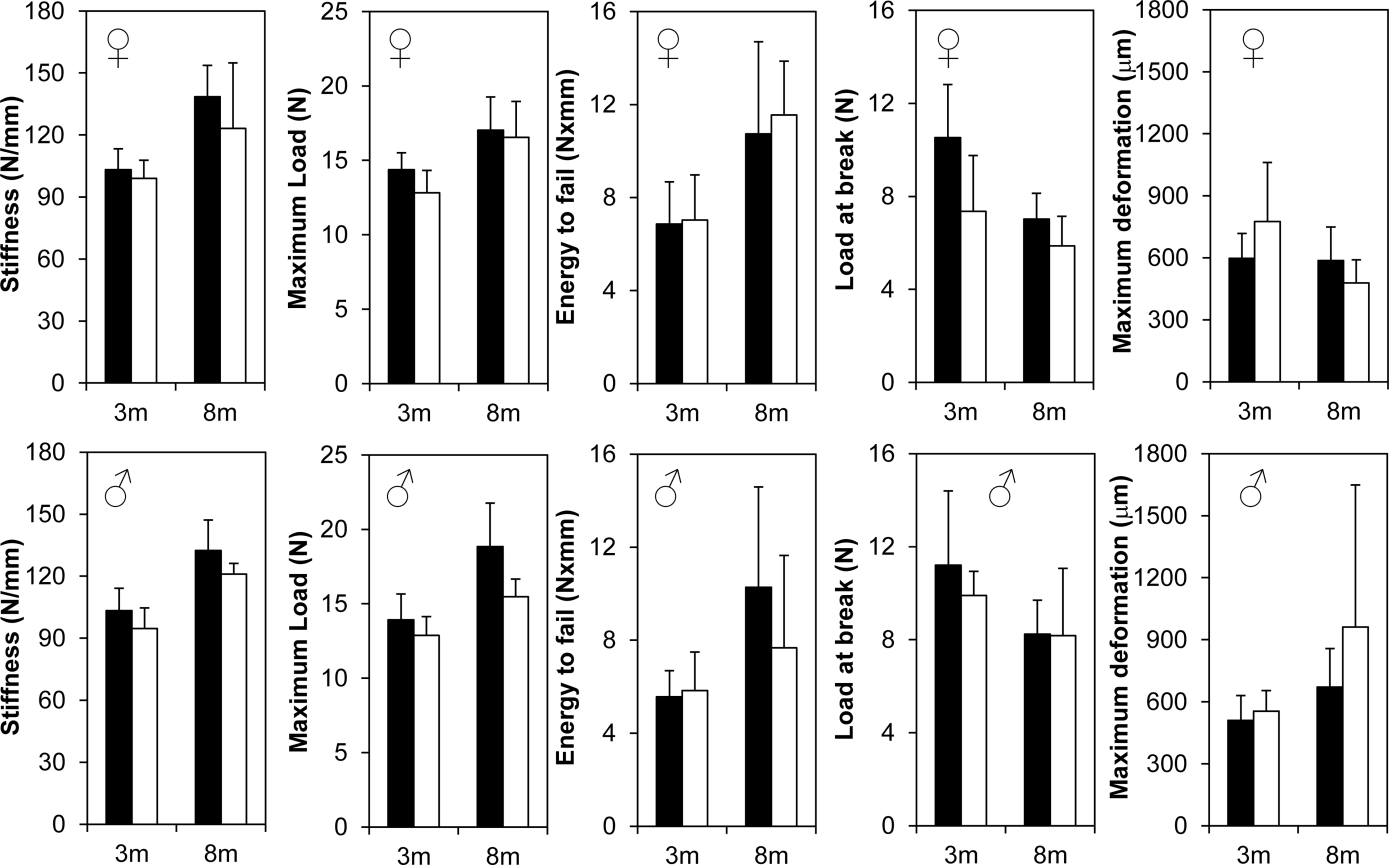
Femur biomechanical analysis on 3m and 8m old female (top row) and male (bottom row) femurs. Femurs were analyzed by the three point bending test. Bones were loaded to failure at the midshaft and mechanical properties were measured from the resulting load vs. displacement curve. White bars represent WT groups and black bars represent KO groups. Values represent mean ± SD at each age point. Group size is n = 5 for all age points except 8m old KO where n = 3. Mechanical property measurements indicate no significant changes at any ages in both genders.

To finely characterize and detect any potential changes in bone mineral density and structure in the *Bril* KO mice, microcomputed tomography (μCT) scans were performed on distal femurs at 6w, 3m and 8m for both males and females. Quantitative trabecular (Fig 4) and cortical bone parameters (Fig 5) were also derived and described. All trabecular parameters remained unchanged in the female KO versus WT littermates except for a transient significant effect detected only at 6w as illustrated by a 25% decreased BV/TV. Correspondingly, there was a 25% increase in trabecular separation and 24% decrease in trabecular number, but no changes in trabecular thickness (Fig 4-top row). In males, no statistically significant differences were detected in all parameters measured. For the cortical bone compartment (Fig 5), again some female and male KO mice displayed transient decreases in some of the parameters, but none were observed systematically across all ages. Females had significantly decreased bone areal fraction at 6w (11%) and 3m (7%), as reflected by a corresponding 18% and 10% decrease in cortical thickness. Male KO mice had a ~10% decrease in bone areal fraction at 6w but not at 3m. A cautionary note is that the group size for 8m males was only 3 animals. As a corroborative complementary technique, a 3-point-bending assay was performed to assess the material properties of the femur at 3m and 8m (Fig 6). Although variability was somewhat greater amongst groups, as reflected by the large standard error bars for data presented in Fig 6, there were no statistical differences in any of the parameters for both genders.

### RT-qPCR gene expression monitoring in humeri

**Fig 7:**
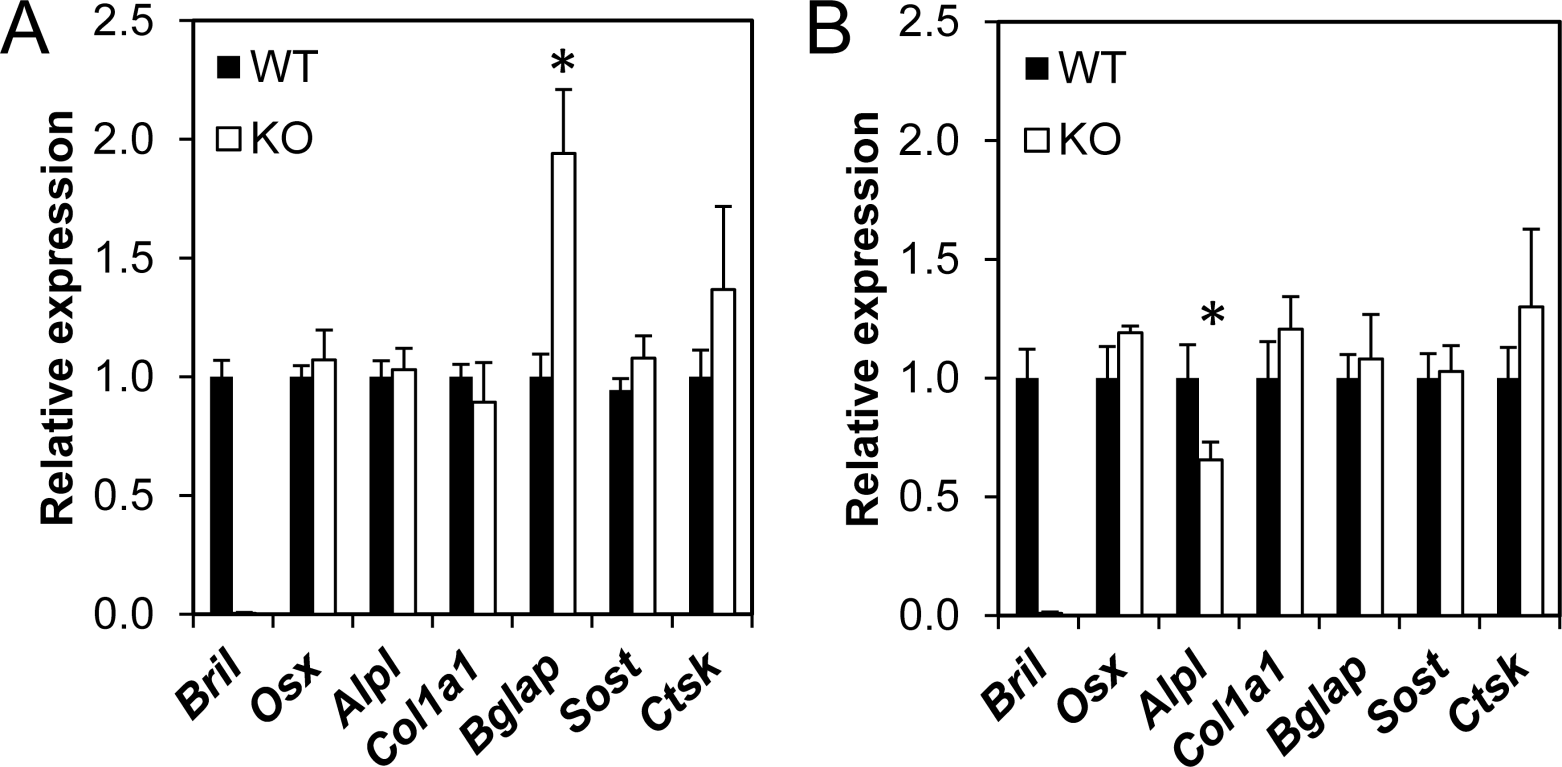
RT-qPCR gene expression monitoring in humeri. Total RNA was extracted from humeri of 6w (A) and 3m (B) WT and KO littermates. RT-qPCR analysis of mRNAs encoding listed genes was performed with respective Taqman probes. Expression levels were normalized to β*-actin* and data are plotted relative to WT values for each gene. Values represent mean ± SEM (n = 6).

We next monitored whether expression of genes representative of osteoblasts (*Osx*, *Alpl*, *Col1a1*, *Bglap*) osteocytes (*Sost*), and osteoclasts (*Catk*) were altered by ablation of *Bril* (Fig 7). Total mRNA was extracted from 6w and 3m-old humeri (n = 7) and used for RT-qPCR with gene-specific Taqman probes. Levels were normalized to β*-actin* and presented as relative to those measured in the WT. The absence of *Bril* expression in the KO samples validated the genotype at both ages (Fig 7A and 7B). At 6w of age, only *Bglap* was significantly changed (2-fold increase) in the KO mice (Fig 7A). At 3m of age, however, *Bglap* levels had normalized and only *Alpl* was significantly altered, being reduced by 35% of WT levels (Fig 7B). No other changes were observed in any of the other genes tested. Expression levels of *Iftm1*, *2*, and *3* also did not differ in calvaria of 6w KO from WT, suggesting no compensatory mechanisms by these members (S4 Fig).

### Measurements of serum markers for bone formation and resorption activity

**Fig 8:**
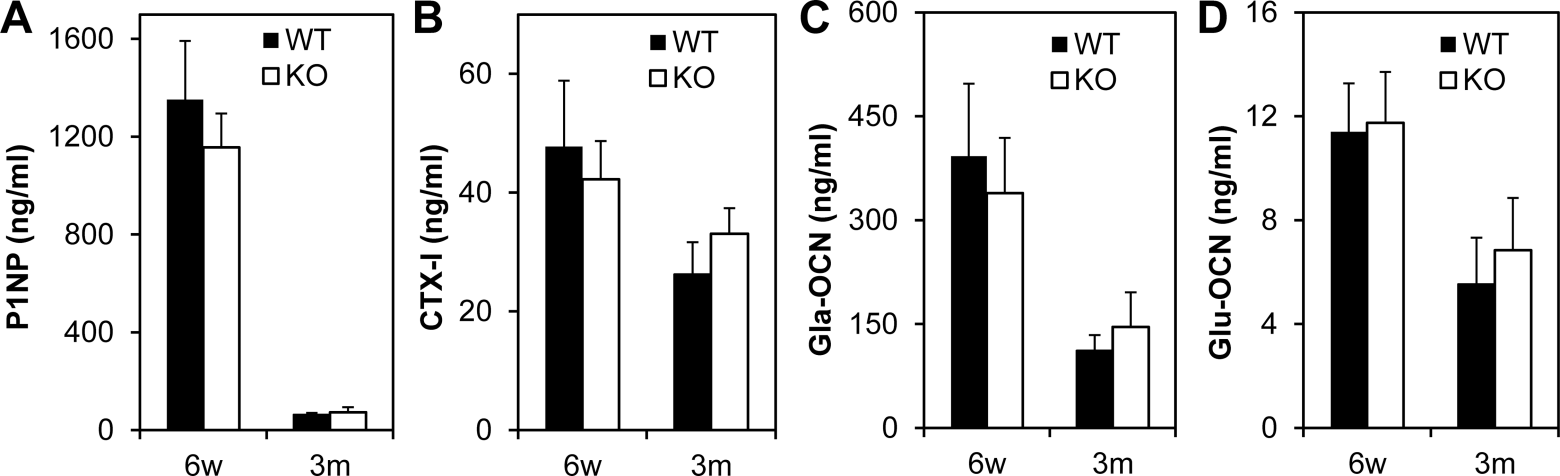
Serum bone turnover measurements in WT and KO males at 6w and 3m. Serum was collected at sacrifice and analyzed for bone resorption and formation using ELISA kits for A) N-terminal propeptide of type I collagen (P1NP); B) C-terminal telopeptide of type I collagen (CTX-I); C) inactive gamma-carboxylated osteocalcin (Gla-OCN); and D) active undercaboxylated (Glu-OCN) osteocalcin. No significant differences were measured for either parameter. Values represent mean ± SD (n = 4).

To further validate if bone formation and resorption parameters were altered in the *Bril* KO mice, blood was collected and serum was processed for analytical biochemistry measurements (Fig 8A and 8B). Elisa were run to assay for markers of collagen type I formation (N-terminal propeptides (P1NP)) and resorption (C-terminal telopeptides (CTX-I)) in actively growing juvenile (6w) and adult (3m) males. As expected, the P1NP levels decreased substantially from 6w to 3m, but there were no statistical changes between the WT and KO groups (Fig 8A). CTX-I levels also slightly decreased from 6w to 3m, but not between WT and KO. Circulating levels of the inactive gamma-carboxylated (Gla-OCN) and active undercaboxylated (Glu-OCN) forms of osteocalcin were also not significantly altered between the WT and KO mice (Fig 8C and 8D). A more extensive biochemistry panel was tested on the serum of 3m males (S1 Table). Among the 16 analytes tested, only alkaline phosphatase (ALPL) activity was slightly decreased in the *Bril* KO as compared to WT (83.8 ± 9.9 versus 100.8 ± 8.2 U/L respectively; mean ± SE (n = 4); p = 0.03). Values for ALPL of both groups were still within the normal range for mice (62–209 U/L). Of note, calcium, phosphorus and glucose also remained unchanged (S2 Table).

### Rodded fracture repair surgeries

**Fig 9:**
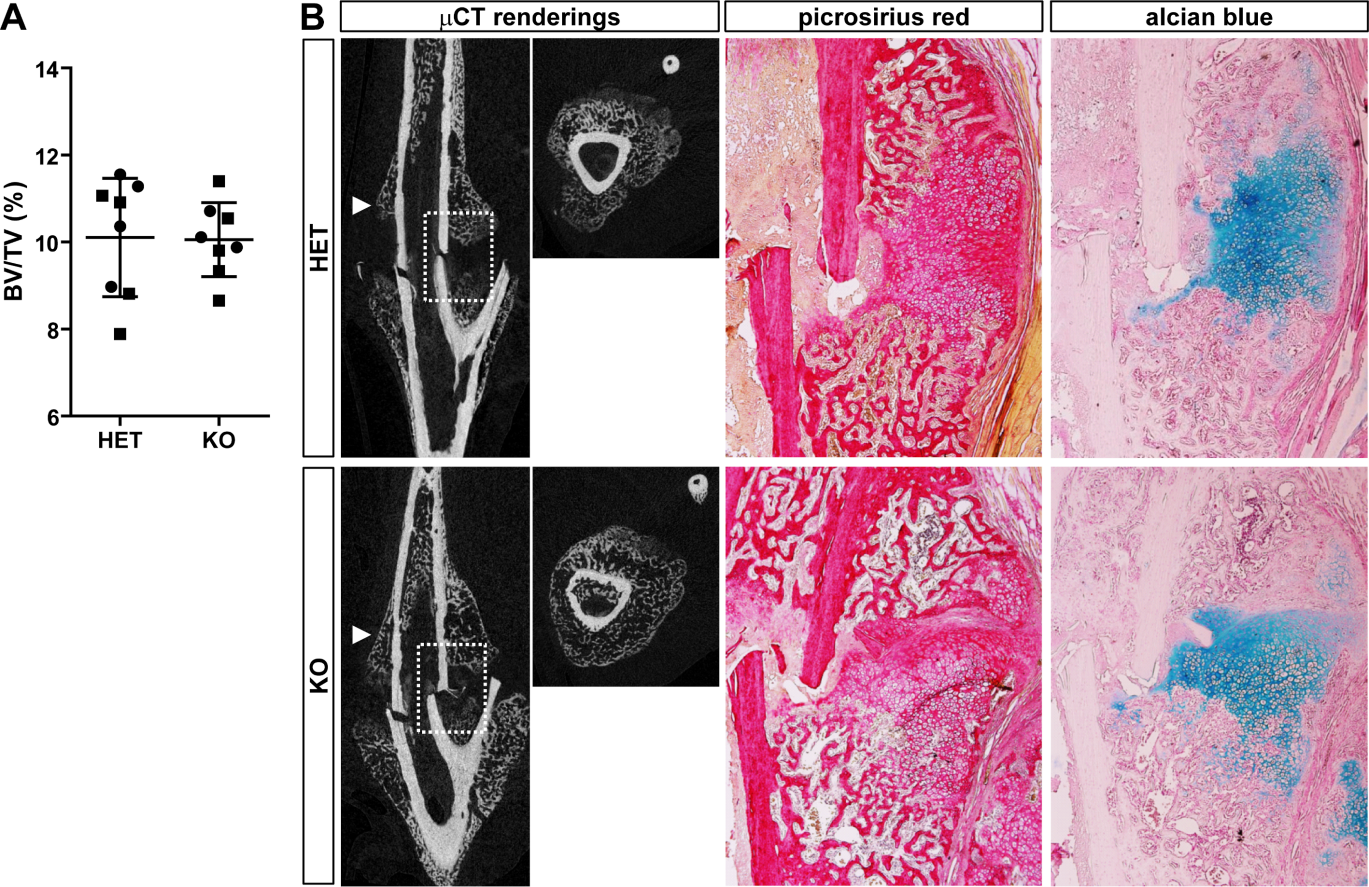
Rodded fracture repair experiment conducted on HET and KO mice at 4m of age. A) Quantitative μCT measurements of the callus area 14-days after a mid-tibial fracture in males and females HET and KO (n = 8 per genotype; ■ males; ● females). The BT/TV percentage was determined using a fixed volume as described in materials and methods. Bars represent mean ± SD. No differences in the BV/TV of the fracture callus were detected between the two groups. B) At left, representative 2-D μCT renderings for the YZ longitudinal and YX trans-axial (pointing arrowhead) views for the HET (top) and KO (bottom). At right, representative histological images corresponding to the boxed area shown in the μCT. Picrosirius red and alcian blue stain for collagen (red) and cartilage proteoglycan (blue) network, respectively. Alcian blue counterstain is nuclear fast red.

Under normal vivarium conditions, bone development and mineralization generally proceeded independent of BRIL. To test the possibility that they would show an altered response upon a challenge, a rodded fracture repair experiment was conducted. The surgical procedure was performed on the left tibia of cohorts of 4m old *Bril* HET and KO mice (8 per group; 4 males and 4 females). Given the absence of a strong skeletal phenotype observed thus far, we decided to collect samples at a single time point, 14-days later, representing a critical stage at which the callus has started to remodel and mineralize. The tibias were processed for uCT and, after decalcification, histological analyses. Quantification of the BV/TV, calculated using a fixed tissue volume as described in Materials and Methods, revealed no significant differences in the mineralized callus area between the HET and KO mice (Fig 9A), nor between males and females. Representative 2 dimension uCT rendering are presented in Fig 9B, with corresponding histological sections stained with picrosirius red (Fig 9C) or alcian blue (Fig 9D). The extent and gross appearance of the extracellular matrix collagen network (Fig 9B) and that of the un-remodeled cartilaginous (Fig 9C) within the callus presented no obvious differences.

### Calcium binding assay

**Fig 10:**
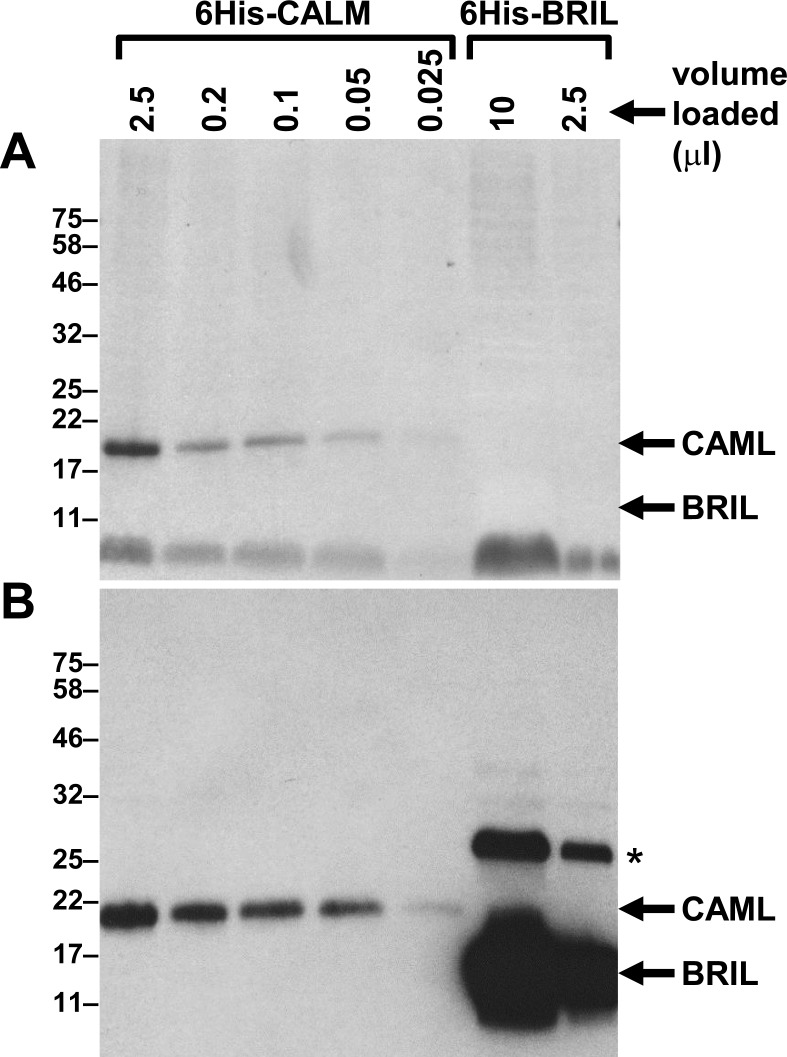
Calcium binding assay on recombinant mouse CAML and BRIL. Bacterial extracts overexpressing 6-histidine-tagged mouse CALM or BRIL were separated on duplicate 8–16% SDS-PAGE. Different volumes were loaded as indicated with BRIL being in excess to that of CALM. Proteins were transferred to PVDF membranes and assayed for ^45^Ca binding by autoradiography (A), or probed for expression with an anti-histidine antibody (B). Position of CALM and BRIL migration are indicated with arrows. Molecular mass markers (kD) are indicated at left. Asterisk point to an oligomeric form of BRIL.

It has been previously proposed that the C-terminal tail of BRIL may possess a potential calcium binding motif. To verify that possibility, recombinant mouse BRIL and calmodulin (CALM) proteins tagged at their N-terminus with a 6-histidine epitope, were produced in bacteria and extracts were tested for their ability to bind calcium using an established procedure [29]. CALM was chosen as a positive control for the assay because its size (17kDa) is comparable to that of BRIL (14kDa). Bacterial extracts were separated on duplicate SDS-PAGE, transferred to PVDF membranes, and probed either with ^45^CaCl_2_ (Fig 10A) or by western blotting with an anti-6-histidine antibody (Fig 10B). Under those conditions, only the band corresponding to CALM within the bacterial extract chiefly bound ^45^Ca (Fig 10A). Although the extracts containing BRIL were loaded in vast excess (10μl) relative to CALM (0.05μl), as detected by western blotting (Fig 10B), there was no evidence of ^45^Ca binding to BRIL.

## Discussion

The full length cDNA cloning for *Bril* was first reported from primitive hemopoietic cells differentiated from ES cell embryonic bodies [30]. It was subsequently identified as part of the *Ifitm* family cluster [31]. The osteoblastic specific expression of *Bril*, however, was first recognized by our group [1] and confirmed by others [2, 10]. At that time, the physiological role played by BRIL in bone had yet to be uncovered. Although *Bril* was identified as the genetic cause of osteogenesis imperfecta [12–15], this is likely due to a dominant neomorphic function unique to the mutant BRIL protein which may have little to do with its natural context [16]. A previously described mouse model with the global deletion of *Bril* pointed to some important, yet intermittent, role during skeletal development and growth, and perhaps even fertility [2]. Transgenic overexpression of BRIL in osteoblast cells also failed to reveal any impact on bone [16]. The current study aimed to further shed light on the contribution of BRIL in osteogenesis *in vivo*, and address whether it serves an essential role in skeletogenesis using a more in depth series of analyses.

Towards this, we generated a global *Bril* KO mouse model by replacing the 2 exons with a nuclear targeted LacZ reporter. Global inactivation of *Bril* was validated by the total absence of detectable transcript and protein. The combined KO/LacZ knockin strategy allowed monitoring sites of *Bril* expression in situ on whole embryos and tissue sections. The surrogate nuclear X-Gal staining mimicked that of endogenous *Bril* expression patterns previously described in mice using in situ hybridization and immunohistochemistry [1, 2, 6]. Earliest X-Gal staining was detected at around E13.5 in the developing bony elements of the jaw and forelimb. This timing corresponds to expression of osterix [32], one of the earliest transcriptional drivers of osteoblastogenesis together with Runx2 [33, 34]. The expression of LacZ at later stages up to E18.5 produced very intense staining in all skeletal elements formed through intramembranous and endochondral processes, which very closely mirrored other models utilizing osteoblast Cre drivers for activation of the Rosa-LacZ floxed reporter [32, 35–37]. Staining over tissue sections, where the majority of blue nuclei-stained cells were associated with bone forming surfaces, corroborated the exquisite osteoblast-specific nature of *Bril* expression, which makes it a useful cell-surface biomarker [1, 2]. Only rare non-osseous sites of expression (nostril and dental pulp/odontoblasts) were observed. Given this tightly regulated expression and specific staining, we propose that this constitutive model could be used for tracking osteoblasts, not only in embryos, but into adulthood as the reporter was still active at 9w of age. Furthermore, the *Bril* regulatory sequences could be exploited to develop new tools, such as Cre-driver lines, which would possess a stricter osteoblast-specific excision potential for floxed gene.

Breeding schemes to generate the experimental animals, whether through intercrosses of HET or KO mice, indicated a completely normal Mendelian inheritance of the KO allele in descendants. These data exclude any unforeseen, direct or indirect, reproductive problems due to the lack of BRIL and contrast with a previously described *Bril* KO model. Indeed, Hanagata *et al*. [2] reported that *Bril* null homozygous mice had difficulty breeding, having an average of 1 pup per litter, with a possible more detrimental outcome for females. In our model, the minor gender skews and differences detected were not in fertility, but rather in females displaying slightly reduced femur length (Fig 3), reduced trabecular bone volume fraction, increased trabecular spacing and number (Fig 4 top vs bottom); and reduced cortical bone area (Fig 5), as compared to males. Additionally, the previous study reported that newborn KO mice had skeletal abnormalities such as reduced long bones length and bends, but overall presented no changes in any bone morphometric parameters [2]. As reflected by alcian blue/alizarin red staining of newborns and the gross appearance of X-Gal stained KO mice and corresponding tissue sections, no noticeable deformities were observed in our model. If present, these potential defects would not be severe enough to cause early lethality as exemplified by the normal survival rate of KO homozygotes. Considering the very robust expression of *Bril* in osteoblasts of embryonic bones [1], it could have been predicted to serve an essential function for skeletal patterning and development, but our data suggest this is not the case. They are also in line with another KO model having a complete deletion of the entire *Ifitm* gene locus [20]. Mice lacking *Ifitm1*, *2*, *3*, *5*, and *6* were found to be viable, fertile and without any apparent abnormal growth despite the crucial role attributed to IFITM1 and 3 in primordial germ cells [38].

To clarify whether BRIL is important for skeletogenesis postnatally, we performed systematic quantitative phenotypic assessment at 3 time points; juveniles (6w), young (3m) and aged (8m) adults. Overall, some statistical significant differences between WT and KO mice were observed in only some of the parameters measured. However, none of these changes were systematically recorded across all age groups and for both genders, but rather reflected transient changes from which the mice recovered. For example, body weight gains for 3m and 8m old mice seemed to be reduced in males but not females. One possible explanation for this could be related to the regulation of whole body energy expenditure controlled by the skeleton-derived hormone BGLAP (osteocalcin) [39]. However, despite a doubling of *Bglap* expression in humeri at 6w in the KO mice, circulating levels of total and active form of the osteocalcin protein were not altered compared to WT. In contrasts, bone length in males remained unchanged at all ages, whereas that of females was reduced by 9% only in females at 6w and 3m, but not at 8m. These changes in bone length were similar though in extent to those previously reported (4–12% decrease) in the KO mice at 8m and 12m [2]. Histological inspection of the growth plate architecture revealed no obvious changes that could account for the slowed bone growth.

At the level of quantitative μCT, for both trabecular and cortical static bone parameters, again some significant changes were observed but not consistently for all ages and genders. Trabecular bone density indicated a 25% deficit in young growing 6w females, which tapered off by 3m and 8m. However, trabecular values did not change in males. Cortical areal bone percentages were also decreased by 10% in both males and females at 6w. None of these changes seemed to translate into significant alteration in bone biomechanical properties. At present it is difficult to reach a unifying explanation for the sporadic changes observed and whether the gender-bias effect is real. Unfortunately, bone microarchitecture analyses by μCT imaging were not analyzed for adult mice in the previous *Bril* KO study [2], so direct comparisons are not possible. Confounding factors, such as the mixed genetic background of the mice and the small group size studied, could have contributed to the subtle consequences of the absence of *Bril* under normal vivarium conditions. Mice of both genotypes also recovered similarly well from a fracture repair surgery performed on tibia. Serum analyses also suggested no major alterations in bone turnover and that osteoclasts resorption activity remained unchanged. No change in gene expression for *Catk* also is indicative of stable steady-state numbers of mature osteoclasts. A subtle yet significant reduction in *Alpl* gene (35%) and serum ALPL activity (17%) was detected but only in 3m old KO. ALPL is a surrogate marker of osteoblast differentiation and an essential enzyme controlling mineralization [40]. The absence of a skeletal phenotype in the ALPL heterozygote KO mice [41], which display a 50% decrease in circulating levels of ALPL, would argue that the small changes caused by the absence of BRIL would not be sufficient to impact overall bone parameters.

Although compensatory mechanisms could be operative to explain the absence of a robust phenotype in the *Bril* KO, they would probably not rely on the other IFITMs as their expression was not significantly changed. In addition, the primary function of IFITM1 to 3 is to block cellular entry and infection against a broad range of viruses [7, 8]. Whether BRIL could participate to such a viral-restriction role in the bone context has not been investigated in details. To our knowledge, only one report examined the comparative effect of different IFITM proteins, including BRIL, at inhibiting viral infection in human lung A549 cells by murine leukemia virus pseudotyped with various virus envelop glycoproteins [42]. In that context, overexpression of BRIL was highly efficacious to prevent infection by pseudovirion harboring Marburg and Ebola viruses’ entry proteins and to a much lower extent by influenza A. Another yet unexplored possibility would be for a bone-specific role of BRIL against arthritogenic alphaviruses like Ross River, Sindbis, and chikungunya viruses. These so-called old-world viruses can cause severe bone symptoms in humans with manifestations, sometimes persistent, comparable to rheumatoid arthritis [43]. Osteoblasts are susceptible to being infected and perturbed by alphaviruses and recently this has been shown to be mediated through osteoblast production of inflammatory cytokines (IL6, RANKL) and consequent activation of osteoclasts and bone resorption [44]. Although *Ifitm3* has recently been shown to be sufficient *in vivo* to delay chikungunya virus-induced arthritis [45], the possible contribution of osteoblasts in that process was not examined. Our current model would represent a suitable system to test this hypothesis.

It has been previously proposed that the C-terminal tail of BRIL possesses a potential calcium binding motif [18, 19]. This is based on the conserved acidic-rich domain present at the C-terminal end of BRIL. Given the topology of BRIL at the plasma membrane of osteoblasts with its C-terminal extremity facing the extracellular milieu, a reasonable assumption could be that it acts through interaction with the mineralizing matrix. An established calcium binding assay, using bacterially expressed proteins showed that BRIL did not bind Ca^2+^. The assay was validated with CALM, a prototypical calcium binding protein which contains 4 helix-loop-helix structures, also referred to as the EF-hand calcium binding motif [46, 47]. It would appear that the acidic C-termini sequence of mouse (DEEDYN) or human (DDADYD) BRIL differs significantly enough from the typical Dx[DN]xDG sequence of an EF-hand motif, especially the spacing residues, not to allow for direct calcium binding. The lack of Ca^2+^ binding would presumably not be caused by the absence of adequate post-translational modifications on recombinant BRIL, as we have previously shown identical migration of bacterial and mammalian BRIL on SDS-PAGE, suggesting at least no glycosylation [3]. It could still be argued, however, that BRIL interacts with the extracellular hydroxyapatite *in vivo* in a cellular environment.

As was originally proposed for other IFITMs [38], another potential role for BRIL would be to serve as a homotypic interface favoring interaction between osteoblasts and promoting synergistic extracellular matrix laying activity over long stretches. This function could be analogous to that of annexins and pannexins, important players for the intercellular communication in bone [48]. Both these roles, however, would be dispensable and not essential for osteoblast function as shown in the present work. Clearly further studies will be needed to unequivocally establish how and if BRIL participates to bone formation and mineralization, or other unsuspected function. Ongoing molecular characterization of the aberrant mutant BRIL in OI type V might provide some informative clues.

## Supporting information

S1 Fig Consecutive staining with X-Gal and picrosirius red of E15.5 Bril KO head demonstrates co-localization of blue-nuclei stained cells and bone surfaces. A composite image of the head is presented at the top, with the respective boxes magnified below (a through i). Arrow in panel f) indicates weak labeling in the nostril region.
(TIF)

S2 Fig Consecutive staining with X-Gal and picrosirius red of E17.5 Bril KO ilium and femur demonstrates co-localization of blue-nuclei stained cells and bone surfaces. Boxed areas of top panels are shown magnified below.
(TIF)

S3 Fig Representative pictures of alizarin red and alcian blue stained 2 day old WT, HET, and Bril KO pups. (TIF)

S4 Fig RT-qPCR gene expression for *Ifitm* family members in 6w old calvaria (mean ± SD, n = 6). (TIF)

S1 Table Cancellous static histomorphometry measurements done on 9-week WT and KO males (mean ± SE, n = 4). (TIF)

S2 Table Blood biochemistry analyses on 4m WT and KO males (mean ± SD, n = 4). (TIF)
