# Supplementary figures and images for: The osteogenic cell surface marker BRIL/IFITM5 is dispensable for bone development and homeostasis in mice
Source: PLoS One. 2017 Sep 7;12(9):e0184568. doi: 10.1371/journal.pone.0184568 (PMC5589259; doi:10.1371/journal.pone.0184568)

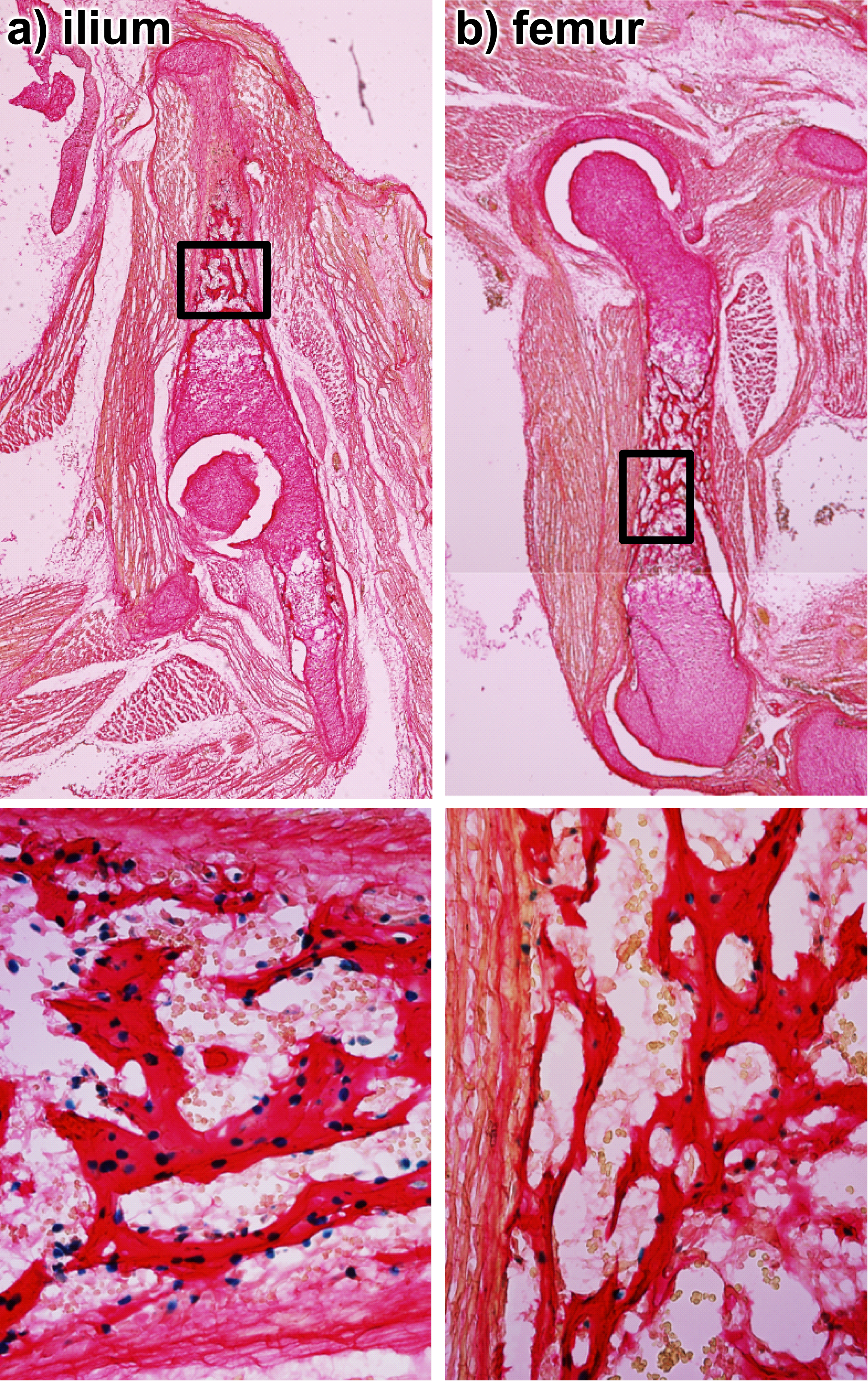

Supplement: S2 Fig — Boxed areas of top panels are shown magnified below. (TIF) [file pone.0184568.s002.tif]

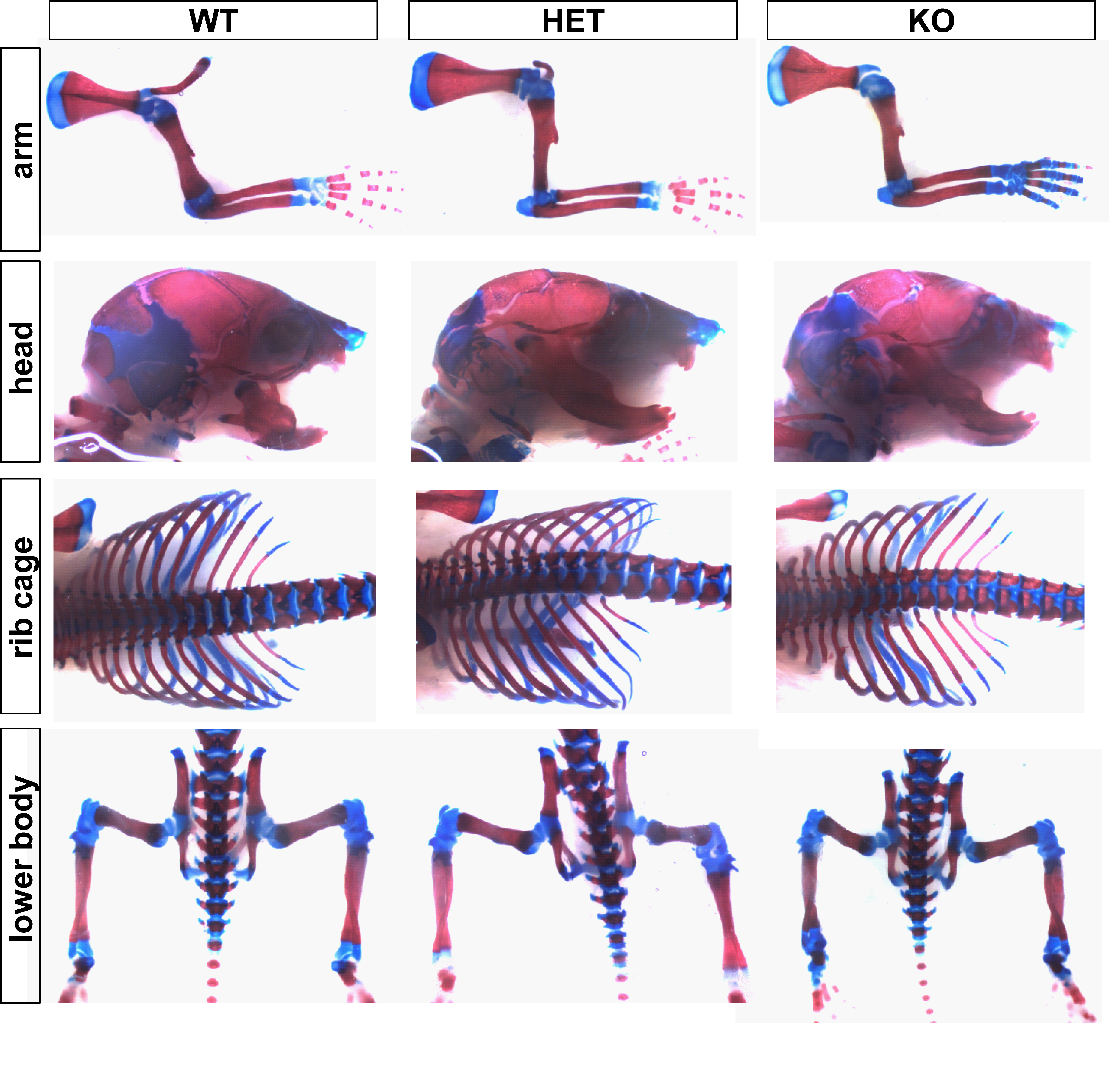

Supplement: S3 Fig — (TIF) [file pone.0184568.s003.tif]

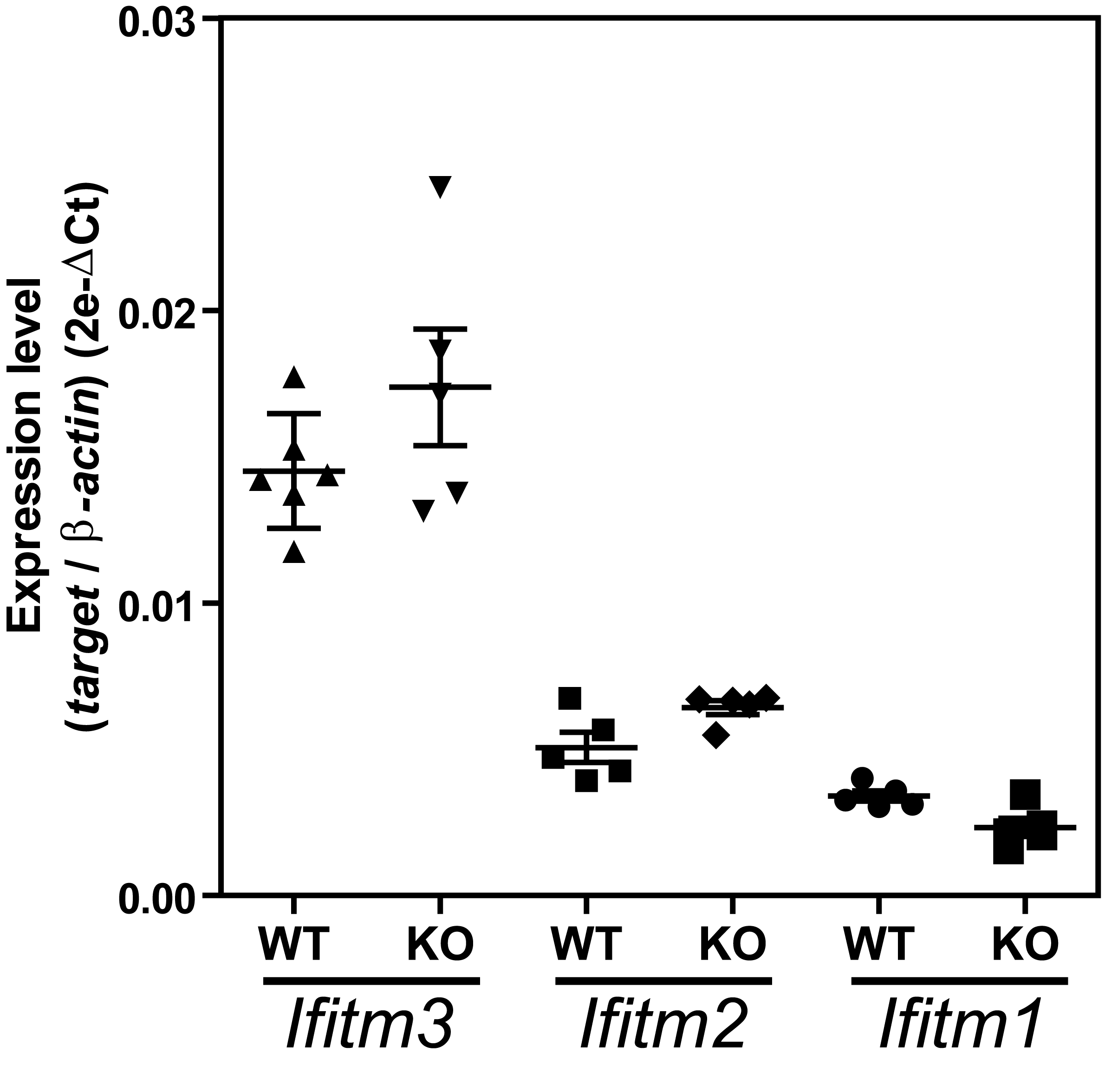

Supplement: S4 Fig — (TIF) [file pone.0184568.s004.tif]

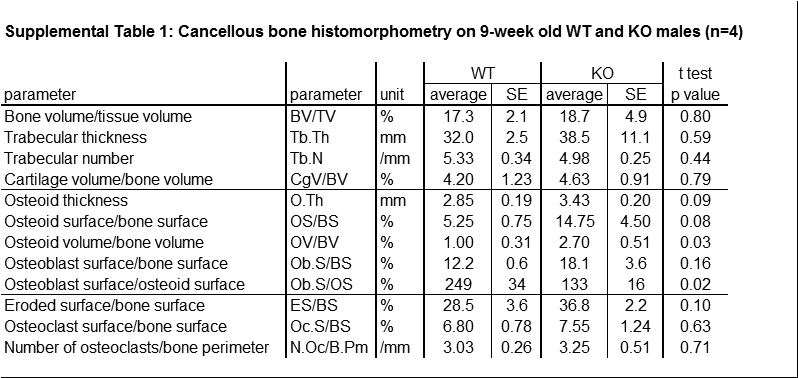

Supplement: S1 Table — (TIF) [file pone.0184568.s005.tif]

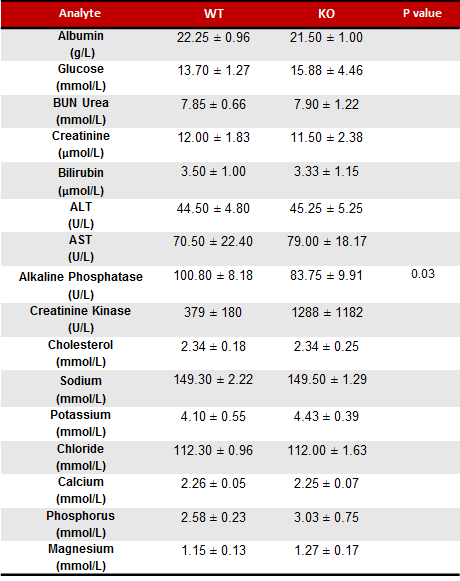

Supplement: S2 Table — (TIF) [file pone.0184568.s006.tif]
